# Rethinking access to psychological treatment protocols in mental health research

**DOI:** 10.1192/j.eurpsy.2025.538

**Published:** 2025-08-26

**Authors:** C. Blithikioti, G. Tomei, F. Visconti, I. G. Gómez, I. A. Cristea

**Affiliations:** 1 General Psychology, University of Padova, Padova, Italy; 2 Psychology, University of Loyola, Seville, Spain

## Abstract

**Introduction:**

Open access to psychological treatment manuals is critical for advancing research and clinical practice, particularly in low- and middle-income countries, where access to mental health care is scarce. Despite growing recognition of the need for freely available manuals to ensure replicability, transparency, and wider dissemination of evidence-based interventions, open and free access to intervention manuals remains limited.

**Objectives:**

We aimed to quantify the availability of protocols and manuals for psychological interventions used in randomized clinical trials (RCTs) for severe mental disorders. This research is part of the broader European Research Council – funded project DECOMPOSE, in which we employ a systematic and reproducible approach for decoding, classifying, and evaluating the active ingredients of psychological interventions.

**Methods:**

Using recent network meta-analyses of RCTs, we collected psychological interventions for psychotic, bipolar, substance use, eating, and borderline personality disorders. We attempted to retrieve intervention protocols and manuals directly from trial publications or their published protocols and referenced manuals. If the protocols or manuals were not accessible, we contacted the study authors to request the materials.

**Results:**

We identified a total of 259 RCTs, but only 18 had published protocols. Of the 71 RCTs pre-registered on platforms such as ClinicalTrials.gov, only 5 provided an adequate description of the psychological treatment components, all of which overlapped with already published protocols. To retrieve missing materials, we contacted 450 authors from 241 RCTs. We received positive responses from 75 RCTs, negative responses from 55 RCTs, and no replies from 100 RCTs. We were not able to retrieve contact information for the authors of 11 RCTs.

Of the 75 positive responses, we obtained the complete requested materials for only 47 trials. In the remaining cases, we were instructed to purchase the manuals (n=11), provided with only partial materials (n=4), or given additional references that were not the full intervention manual (n=13). Negative responses included the trial being too old or no authors’ access to the materials (n=22), commitment to send the materials without further follow-up (n=8), suggesting the paper as the sole available resource (n=11), and various other reasons (n=14)

**Conclusions:**

Our findings reveal a significant lack of freely available intervention manuals, limiting the implementation and replicability of psychological treatments. Coordinated action is needed to ensure open access to these materials for more replicable research, wider dissemination of results, and improved access to evidence-based mental health care.

**Disclosure of Interest:**

None Declared

